# Validation of an Enzyme Immunoassay to Measure Faecal Glucocorticoid Metabolites in Common Brushtail Possums (*Trichosurus vulpecula*) to Evaluate Responses to Rehabilitation

**DOI:** 10.3390/ani12131627

**Published:** 2022-06-24

**Authors:** Holly R. Cope, Tamara Keeley, Joy Keong, Daniel Smith, Fabiola R. O. Silva, Clare McArthur, Koa N. Webster, Valentina S. A. Mella, Catherine A. Herbert

**Affiliations:** 1 Sydney School of Veterinary Science, University of Sydney, Camperdown, NSW 2006, Australia; valentina.mella@sydney.edu.au; 2 School of Agriculture and Food Sciences, University of Queensland, Gatton, QLD 4343, Australia; t.keeley@uq.edu.au; 3 School of Life and Environmental Sciences, University of Sydney, Camperdown, NSW 2006, Australia; joy.keong@hotmail.com (J.K.); dansmiff.ds@gmail.com (D.S.); fabiola.silva@sydney.edu.au (F.R.O.S.); clare.mcarthur@sydney.edu.au (C.M.); catherine.herbert@sydney.edu.au (C.A.H.); 4 Department of Biological Sciences, Macquarie University, North Ryde, NSW 2109, Australia; koa.webster@gmail.com

**Keywords:** captivity, corticosterone, cortisol, wildlife rehabilitation, 11-oxoaetiocholanolone, dermatitis, wildlife rescue, enzyme immunoassay, EIA

## Abstract

**Simple Summary:**

Little is known about how exposure to novel stimuli during rescue and rehabilitation could affect the physiology of native wildlife. We investigated this question in a species commonly rescued for rehabilitation, the common brushtail possum (*Trichosurus vulpecula*). Glucocorticoids (major hormones involved in stress responses) are metabolised in the body and excreted in the form of faecal glucocorticoid metabolites, which can be measured as a way of evaluating the response of animals to potential stressors. Comparing five enzyme immunoassay options, we found that the 11-oxoaetiocholanolone (abbreviation: 72a) EIA was the most suitable for measuring these metabolites in brushtail possums. This assay was then used to measure faecal glucocorticoid metabolite concentrations in 20 possums during rehabilitation. The probability of a physiological “stress” response occurring within five days of a potentially stressful event was about 50%, regardless of the type of event. There was a high level of variation in hormone profiles between possums. Our study has demonstrated that injured and orphaned possums show detectable changes in faecal glucocorticoid metabolites during captivity and rehabilitation, and has identified events that can induce a physiological response in some individuals. This is the first step toward understanding the relationship between these responses during rehabilitation and survival.

**Abstract:**

Volunteer wildlife rehabilitators rescue and rehabilitate thousands of native animals every year in Australia. However, there is little known about how exposure to novel stimuli during rehabilitation could affect the physiology of wildlife. We investigated this question in a species that commonly enters rehabilitation, the common brushtail possum (*Trichosurus vulpecula*). We evaluated five enzyme immunoassays (EIA) to determine the most suitable for measuring faecal glucocorticoid metabolites (FGM) as a proxy for evaluating the response of brushtail possums to potential stressors during rehabilitation. An adrenocorticotrophin hormone (ACTH) challenge was conducted on wild-caught possums to determine the best-performing EIA based on the successful detection of FGM peaks in at least two of three possums. While a number of assays met these criteria, the 11-oxoaetiocholanolone (abbreviation: 72a) EIA was selected as it had the largest amplitude of change in response to the ACTH challenge. This assay was then used to measure FGM concentrations in 20 possums during rehabilitation. There was high variation in baseline FGM concentrations and response to captivity between possums. Significant changes in FGM levels were detected in most possums during captivity, but were not reliably associated with potentially stressful events that were identified by rehabilitators. The probability of an FGM peak occurring within five days of a potentially stressful event was about 50%, regardless of the type of event. Our study has demonstrated that injured and orphaned possums show changes in FGMs during captivity and rehabilitation and has identified events that can induce a physiological response in some individuals. We recommend that research now focus on the relationship between these responses during rehabilitation and pre- and post-release survival.

## 1. Introduction

Thousands of native animals are rescued and admitted for rehabilitation by volunteer wildlife rehabilitators every year in Australia [1,2,3]. The number of native wildlife requiring rescue and rehabilitation has been increasing in recent years, with the most common causes for rescue including ‘collisions with vehicles’, ‘abandoned/orphaned’ and ‘unsuitable environment’ [1,4,5,6]. Over a six-year period from 2013 a total of 469,553 individuals from 689 species were reported to have been rescued in the Australian state of New South Wales (NSW) alone: 53% were birds, 34% mammals and 13% reptiles [1]. Wildlife rehabilitators and veterinarians invest a large amount of time and financial resources into rehabilitating sick and injured wildlife [7,8,9], aiming to relieve pain and suffering, and ultimately release the animal back to the wild [10,11]. However, bringing wild animals that have recently experienced a stressful event or morbidity into captivity could compound existing pressures on their physiology and health through exposure to additional stressors. Such stressors could include changes in their environment, diet, and interactions with humans and domestic animals or other wildlife patients, which they may perceive as threats or even predators [12,13,14,15].

A stressor is an event or stimulus that triggers a physiological response in the animal mediated by the hypothalamic–pituitary–adrenal (HPA) axis with the goal of resuming homeostasis, which can be accompanied by behavioural and autonomous nervous system responses [16,17]. The hypothalamus secretes corticotrophin releasing hormone (CRH) which stimulates the anterior pituitary to release adrenocorticotrophic hormone (ACTH), triggering the release of glucocorticoids (GCs) from the adrenal gland into the circulatory system [18]. The elevation of GCs in response to an acute stressor is transient and can be beneficial, activating gluconeogenesis and the mobilisation of energy for a fight-or-flight response [18,19,20]. A chronic stressor causes a long-term change in GC secretions and creates a physiological state where negative feedback functions are impaired with potential knock-on effects on immune function and health [14,16,21,22]. For these reasons, GCs are often referred to as stress hormones, although their biological effects are broader than this [23,24]. Animals experiencing chronic stress may demonstrate reduced reproductive rates, immunosuppression, loss of body mass [17,25,26,27,28], and possibly reduced survival [29,30,31]. It has been argued that the negative impacts of chronic stress may manifest pathologically for animals in the laboratory but not in animals under chronic stress in their natural environment; with the latter still responding adaptively to promote fitness [32].

Circulating GCs in the bloodstream are metabolised into a range of molecules in the liver and gut which are excreted in faeces after a variable lag time. Faecal glucocorticoid metabolites (FGMs) are therefore a useful proxy measure for circulating GCs and can be quantified using an appropriate enzyme immunoassay (EIA) for the relevant metabolites [33,34]. This non-invasive method has the advantage that samples can be collected without causing distress to the animals, as can be the case for the collection of blood or saliva [17,18]. GC concentrations in non-invasive biological samples, such as faeces, urine and fur, are increasingly being used to evaluate physiological responses to stressors in animals, including livestock and wildlife [17,34,35,36]. FGM assays require validation to determine the most suitable metabolite to measure in the species [34,37]. This is often achieved by artificially stimulating the HPA axis with exogenous ACTH (a so-called ACTH challenge) and assessing which assays successfully detect the transient increase in FGMs shortly thereafter [34,38]. This approach is also useful in determining the lag time from when an animal is first exposed to a stressor to the first increase in excreted FGMs [34,37]. This lag can be up to 24–48 h in marsupials [39,40,41] and differs between species due to varying species-specific metabolism and excretion [34].

Common brushtail possums (*Trichosurus vulpecula*) (hereafter, brushtail possums) are in the top 10 most-rescued species in NSW, Australia, with 5273 individuals rescued in the 2019–2020 financial year [1,6]. This endemic marsupial is widely distributed across Australia and is a resilient and adaptable species currently listed as Least Concern on the IUCN (International Union for Conservation of Nature) Red List, yet its overall population is decreasing [42,43]. Given the large number of animals entering rehabilitation, it is vital to identify aspects of the rescue and rehabilitation process that could pose animal welfare concerns or be targeted to reduce unnecessary distress and improve outcomes. The extent to which stress can impact the success of treatment and rehabilitation of wildlife is not known [44,45], however, some human medical studies have found that reducing stress improved treatment outcomes for cardiac patients [46,47,48], and it is known that stress can significantly alter wildlife immune function and survival [49].

The relationship between captivity and the physiological response to stressors, as evidenced by changing GC concentrations, has been studied in several species [13,50,51,52], yet it remains under-investigated for wildlife that enter rehabilitation. As animals that enter captivity for rehabilitation are already compromised, the physiological impacts of captivity may vary compared to healthy captive animals, hence, captivity may further compound their already compromised fitness. A recent study found that hedgehogs (*Erinaceus europaeus*) in rehabilitation had higher levels of faecal corticosterone metabolites and saliva corticosterone than wild hedgehogs [53], highlighting that GC responses could be associated with certain factors related to rehabilitation. A pilot study of koalas (*Phascolarctos cinereus*) in rehabilitation found that faecal glucocorticoids increased on days when a stressor was recorded in two of three studied koalas [45]. However, there is a lack of data for other animals that frequently enter rehabilitation, such as the brushtail possum.

This study aimed to investigate the physiological response of brushtail possums to captivity associated with rehabilitation or hand-rearing. However, an appropriate FGM EIA has not been validated in this species [34,54]. Hence, the aims were two-fold: (i) to determine the most appropriate EIA to detect changes in FGMs in brushtail possums; and (ii) to use this assay to identify stressors during rehabilitation that trigger a change in GC concentrations and may therefore influence rehabilitation success. Four wild caught possums were temporarily housed in captivity and exposed to an ACTH challenge, and five different FGM assays were evaluated to determine the most suitable (best) assay for this species. The best assay was then used to evaluate changes in FGM in 20 captive brushtail possums undergoing rehabilitation to test for a relationship between temporal changes in FGM concentration and potential stressors, or with the reason for rescue, time in rehabilitation, age, and sex.

## 2. Materials and Methods

### 2.1. Validation of Enzyme Immunoassays

#### 2.1.1. Animal Capture, Handling, and Husbandry

Trapping was carried out over two consecutive nights (16–17 April 2018) at Macquarie University North Ryde campus (33°46′07.7″ S 151°06′47.5″ E). Eighteen Tomahawk live cage traps (65 × 24 × 24 cm) were covered with hessian and plastic bags and baited with half a peanut-butter sandwich and a quarter slice of apple. The traps were strategically placed around the campus on flat ground approximately one hour before sundown (16:30 to 17:30 AEST). All of the traps were checked 6–7 h after being set (23:30 to 00:30 AEST). Two female brushtail possums carrying back-riding young were immediately released at their site of capture. Adult brushtail possums in a healthy condition (body mass > 1.5 kg) of either sex were transferred from the site of capture in the hessian covered trap (maximum 5 min in transport) to their temporary housing for the challenge.

The brushtail possums (*n* = 3 adult female, *n* = 1 adult male) used for the ACTH challenge were housed at the Macquarie University Fauna Park in individual wire enclosures (1.4–2.2 m wide, 1.8–1.9 m high, 4.2–5.0 m long) for nine nights. PIT tags (Biomark GPT12 pre-load sterile) were implanted under the skin between the shoulders for the identification of possums that were not previously tagged. The possums were housed individually in randomly assigned enclosures with an empty pen in between to reduce negative interactions that could affect their stress response. Plywood sheets were attached to the back half sides of the enclosures to separate them, and concrete flooring and steel roofing provided weather protection. The front half of the enclosure was unsheltered, exposed to adjacent pens, and laid with mulch. Tree branches were provided as enrichment, and wooden nest boxes were supplied. Food and water were provided daily (midday) ad libitum in bowls placed on top of a large plastic tub (60 × 43 × 33 cm) facing away from adjacent pens. This was undertaken to encourage the brushtail possums to leave their nest boxes to eat and drink as they would in the wild. A mixed diet of vegetables (celery, carrot, green beans, and snow peas), fruit (apple, banana, apricot, and pear), nuts (walnuts and almonds) and local native foliage was given. A small amount of supplementary food (peanut butter on whole-wheat bread, cat food, rabbit lucerne pellets, oats) was also given as enrichment. Food intake was recorded daily (in grams) as the amount of food eaten and the amount remaining from the previous day. The brushtail possums were weighed on entry into captivity (day zero), day two, day five, and upon release by suspending them in a hessian handling bag from a spring balance (Pesola Macroline 80005). Any individual showing poor food intake or a 7–10% reduction in body mass in the first 3–5 days was released from captivity at their original capture site (*n* = 1, see Section 3).

#### 2.1.2. Adrenocorticotrophic Hormone (ACTH) Challenge

On the night of capture (day zero), each brushtail possum (*n* = 4) received an intramuscular injection to the thigh of 1 mL of synthetic ACTH, Synacthen (Novartis Australia, North Ryde, NSW, Australia, 250 ug/mL), using a sterile 19 mm, 22-gauge Terumo needle. Fresh faecal scats were collected from each enclosure on day zero as a baseline measure. Samples were collected up to twice daily on days one through nine and stored at −20 °C. The ground was swept clean after each sample collection.

Fresh faecal samples (approximately 5–10 fresh (wet) pellets each, from a discrete pile) were collected from 3 of 4 individuals prior to administration of ACTH (Day 0), from all possums within 24 h of ACTH administration, and on each day in captivity. Brushtail possums reportedly produce an average of 70 faecal pellets per day [55]. One faecal sample from each day in captivity for each brushtail possum was randomly selected for steroid extraction (a total of 34 faecal samples; Female 1 = 10, Female 2 = 10, Female 3 = 4, and Male = 10 samples).

#### 2.1.3. Steroid Extraction and Analysis

To quantify FGM levels from faecal samples, frozen samples were heat dried for approximately 18 h in a laboratory oven (Qualtex Thermostat) set at 60 to 70 °C. They were then crushed using a mallet and sieved through fine mesh. The sieved faecal powder was stored in vials at −20 °C until steroid extraction took place. For steroid extraction, the samples were weighed (0.1 g; range 0.08 g to 0.12 g) and transferred into test tubes, then 2.5 mL of 80% methanol was added to each tube. The tubes were vortexed for 15 s and then shaken on an orbital shaker (Thermo Scientific, Waltham, MA, USA) at 210 rpm for 1 h at room temperature before being centrifuged at 2500 g for 15 min. The supernatant was transferred into labelled glass vials and stored at −20 °C.

The Arbor Assays corticosterone EIA mini-kit (ISWE007; Arbor Assays^®^, Ann Arbor, MI, USA) (hereafter referred to as AA-corticosterone) was run as per the manufacturer’s instructions [56]. It has a cross-reactivity of 100% for corticosterone, 0.22% for cortisol, 0.14% for progesterone, 0.09% for testosterone, 0.08% for cortisone and 0.03% for 17β-Estradiol (as per the manufacturer). Faecal extracts were diluted to 1:20 in assay buffer prior to analysis.

The corticosterone assay, with components produced by Coralie Munro (CJM006; University of California Davis, Davis, CA, USA) (hereafter referred to as CJM006) was run as reported elsewhere with faecal extracts diluted to 1:16 in assay buffer prior to analysis [37]. The polyclonal CJM006 antibody has a cross-reactivity of 100% with corticosterone, and the remainder are as reported elsewhere [57].

The 11-Oxoaetiocholanolone (72a) EIA produced in Vienna (University of Veterinary Medicine, Vienna, Austria) has previously been used in broad-spectrum FGM investigations [58]. The assay was run as described in the protocol by Palme and Mostl [59], where cross-reactivities were identified, with faecal extracts diluted to 1:14 in assay buffer prior to analysis. The 72a EIA was the only group-specific assay utilised in the study (measuring the group of cortisol metabolites, 11,17-dioxoandrostanes).

The cortisol EIA (hereafter referred to as R4866) was originally developed by Coralie Munro (R4866; University of California Davis, Davis, CA, USA) and was used as described previously [60] with faecal extracts diluted 1:8 in assay buffer before analysis. The cross-reactivity of the R4866 antibody was 100% with cortisol, and the remainder are as reported elsewhere [61].

The Arbor Assays cortisol mini-kit EIA (ISWE002, Arbor Assays^®^, Ann Arbor, MI, USA) (hereafter AA-cortisol) was run as per the manufacturer’s recommendations with faecal extracts diluted to 1:12 in assay buffer prior to analysis. It has a reported cross-reactivity of 100% for cortisol, 42.08% for dehydocortisol, 26.53% for cortisone, 4.1% for dexamethasone, 3.37% for prednisone, 0.35% for corticosterone and 0.18% for desoxycorticosterone (as per the manufacturer).

EIAs in the validation analysis were evaluated using a 450 nm filter on an ELx808 BioTek microplate reader using Gen5 Software at the time of plate reading, and the intra- and inter-assay coefficients of variation were <10% and <15% respectively for all assays. All samples from an individual were run on the same plate. Parallelism was demonstrated for serial dilutions of pooled samples relative to the standard curve for each EIA (72a: R^2^ = 0.95, AA-cortisol: R^2^ = 0.95, AA-corticosterone: R^2^ = 0.98, R4866: R^2^ = 0.99, CJM006: R^2^ = 0.96).

### 2.2. Assessment of FGM Levels of Possums in Care

#### 2.2.1. Sample Collection

Volunteer wildlife rehabilitators from the Wildlife Information, Rescue and Education Service (WIRES) branches in and around the Greater Sydney region in NSW were recruited to take part in the study. Rehabilitators were provided with a sampling kit containing plastic zip-lock bags, labels, gloves, and data sheets. They were instructed to collect fresh scat samples from animals on arrival into care, followed by collections every day for a week, then once or twice a week for individually housed possums. The samples were kept frozen to preserve the integrity of the faecal constituents [62]. Rehabilitators were asked to record potentially stressful events during care, such as moving cage or administration of medication. Stressful events were therefore subjectively selected by rehabilitators when observed.

A total of 221 samples were analysed from 20 individual brushtail possums. The possums were classified based on the length of care and severity of ailment as (a) orphan: juvenile possums hand-reared by a rehabilitator; (b) short-term: possums that were in care for less than 14 days and did not suffer from a long-term disease or injury (e.g., infection or broken bone) and (c) long-term: possums that were in care for more than 14 days or suffered from a long-term disease or injury. In each classification, the possums were then divided by sex (male or female) and by life stage (juvenile, sub-adult, or adult). Juveniles were still dependent on their mother or wildlife rehabilitator, sub-adults were no longer dependent on their mother but were not sexually mature, and adults were independent and sexually mature. The 20 possums in this study included nine orphans (four juvenile males, five juvenile females), two short-term individuals (two adult females), and nine long-term individuals (two sub-adult females, one unknown sub-adult, two adult females, three adult females carrying young and one adult male).

#### 2.2.2. Steroid Extraction and Analysis

Scat samples from four of the 20 possums in rehabilitation (IDs 1845, 5862, 8295 and 3215) were sent to the University of Queensland, Gatton, for extraction and analysis to initially confirm that the 72a EIA would detect FGM changes in possums during rehabilitation (i.e., without the synthetic ACTH injection). The extraction protocols described above for the ACTH challenge samples were followed. The samples from the remaining 16 possums in rehabilitation were analysed at The University of Sydney and the extraction protocol was modified slightly due to an increased amount of faecal matter available; in brief, the faeces were dried as described above, weighed at 0.2 g (range 0.18 g to 0.22 g) and extracted with 5 mL of 80% methanol. EIA analyses for all 20 possums’ samples followed the above protocol for 11-Oxoaetiocholanolone (72a) EIA and were read at 450 nm using a BioTek^®^ 800 TS Absorbance Reader and analysed with a 4-parametric logistic fit using MyAssays Analysis Software Solutions (www.myassays.com (accessed on 23 October 2019)). The mean intra- and inter- assay coefficients of variation were 18.3% and 12.6%, respectively. All of the samples from an individual were run on the same plate.

### 2.3. Data Analysis

All statistical analyses were completed in R version 4.0.5 [63]. Parallelism was tested between a serial dilution of pooled faecal extracts and the standard curve using a linear regression. An iterative baseline approach was used to analyse individual possum FGM profiles with the R package ‘hormLong’ [64]. In this method, the mean concentration of the target hormone for each individual is calculated and concentrations exceeding the mean plus 1.5 standard deviations (SD) are removed. The mean is then recalculated multiple times until all remaining concentrations are within the threshold and are thus considered to be baseline values. The values that exceed the final mean plus 1.5 SD (referred to as the baseline threshold) are considered peaks in hormone concentration [37]. We chose 1.5 SD for this small dataset, as it will detect smaller peaks for all individuals [64]. The iterative baseline method did not identify valid peaks for some of the ACTH challenge sample sets due to the limited number of baseline samples compared to elevated samples and the overall small number of samples. Therefore, for the ACTH challenge only, concentrations greater than the first sample plus 1.5 SD (using SD of all concentrations for one individual) were classified as peaks and concentrations lower than this value were classified as baseline (see Figure S1 for comparison of baselines calculated using each method). The times taken for an individual to reach a significant rise in FGMs after ACTH administration and an overall maximum peak in FGMs were calculated using recorded sample dates and times.

The data were highly unbalanced across age (juvenile, sub-adult, or adult), sex (male or female) and type of care (orphan, short-term or long-term) groups. Therefore, we tested for effects of age, sex, and type of rehabilitation on baseline threshold using separate ANOVAs with subsets of data that had sufficient sample sizes as follows: adult females (short-term versus long-term rehabilitation); long-term females (adult versus sub-adult age); orphan juveniles (male versus female sex); all females (short-term, long-term, and orphan types of rehabilitation); all females (adult, sub-adult, and juvenile ages).

Events that were identified by rehabilitators as potential stressors during the sampling period were categorised into Enter Rehabilitation and Other Events. We combined all events other than entering rehabilitation (see details in Section 3.2.1.) into a single category because the low sample sizes (usually <5 per type) prohibited considering them separately in the statistical analyses. We then tabulated the two event categories against the presence or absence of a change in FGM levels thereafter. Responses were defined as a peak in FGM concentration within five days after the event, based on the lag between ACTH exposure and peak FGM observed in the ACTH challenge study. If there was no response five days after the potential stressor it was identified as a non-response. We tested for the fixed effect of event type on the probability of an FGM response occurring using generalised linear mixed models with a binomial distribution and logit link function from the lme4 package in R. To account for a lack of independence between samples we initially included Possum ID as a random factor. However, a comparison using Akaike Information Criterion (AIC) values of the mixed model versus the same model including the fixed factor only showed that including the random factor was overfitting, so our final model included the fixed factor of event type only. As the effect of event type was not significant (see Section 3) we calculated the probability of an FGM response to any event using the null model.

## 3. Results

### 3.1. Validation of Enzyme Immunoassays

Four brushtail possums were successfully trapped and brought into captivity over two trapping sessions, including one male and three females. One female (Female 3) was released from captivity on day four due to declining weight (6.4% body mass loss) and poor food intake. The results for this possum are presented, but not included in the EIA validation analysis due to lack of sample days.

**Figure 1:**
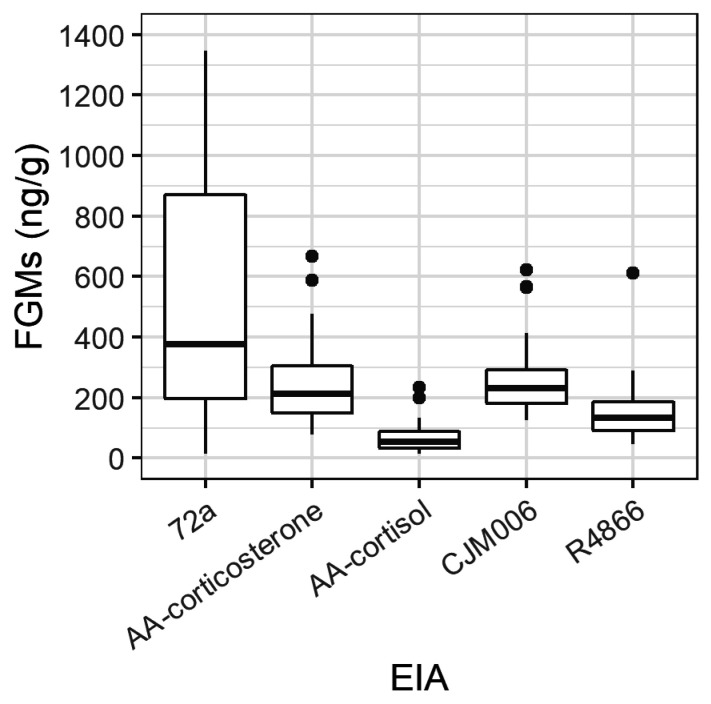
Boxplot of faecal glucocorticoid metabolite (FGM) concentrations (ng/g) as detected by the enzyme immunoassays 72a, AA-corticosterone, AA-cortisol, CJM006 and R4866 for all four brushtail possums across 9 days in captivity following the ACTH challenge (including the pre-challenge scat on day zero; boxplot depicts the median (dark line), two hinges (first and third quartiles), two whiskers (extending from the hinges to the smallest and largest values no further than 1.5 × inter-quartile range) and dots (outlying values)).

**Figure 2:**
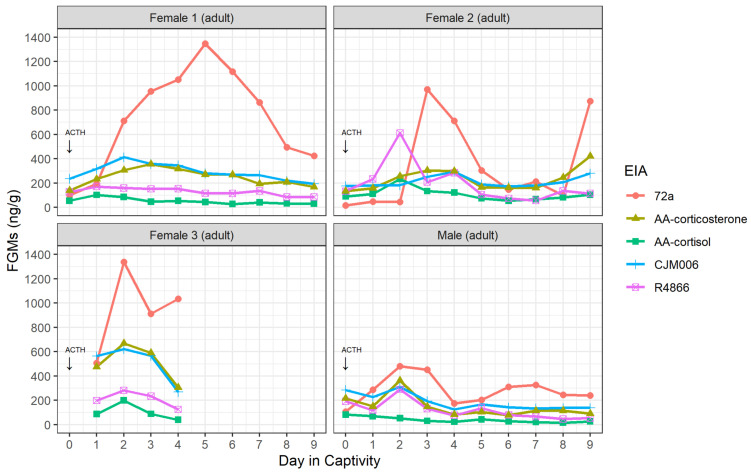
Faecal glucocorticoid metabolite (FGM) concentrations (ng/g) over 9 days (between 16th and 26th April 2018) in captivity for brushtail possums (Females 1, 2, 3 and Male) as detected by the enzyme immunoassays 72a, AA-corticosterone, AA-cortisol, CJM006 and R4866 (note: ACTH was administered on the night of capture as indicated by an arrow within 24 h of the first faecal sample collection).

All five EIAs detected peaks in at least two of three possums (Table 1; Figure S1). The detected peaks ranged in concentration from 83 ng/g (AA-cortisol, Male) to 1347 ng/g (72a, Female 1) amongst the five EIAs and three possums, and the 72a EIA detected the greatest range in FGM concentrations (Figure 1). The 72a EIA showed the greatest increase and magnitude of change in FGM in response to the exogenous ACTH for all possums (Figure 2). FGM increases above baseline occurred following the ACTH challenge for all four possums within 7–55.5 h (Figure 2; Table 1). The time taken for maximum peak in FGMs post ACTH administration varied between possums and EIAs (Table 1).

### 3.2. Assessment of FGM Levels of Possums in Rehabilitation

#### 3.2.1. Sample Collection and Volunteer Response

Six rehabilitators from three WIRES branches (North West, Northern Beaches and Sydney South) provided samples from 34 possums. However, only 20 possums were included in this study due to insufficient sample numbers for 14 possums (≤ four samples). Rehabilitators described 10 event types that may have induced distress in the possums, including entry into rehabilitation, moving cages, escape from the cage, the introduction of new animals, administration of oral medicines, injecting medicines, handling, removing the cover from the cage, another possum riding their back and separation from buddy animals.

#### 3.2.2. FGM Concentration

**Figure 3:**
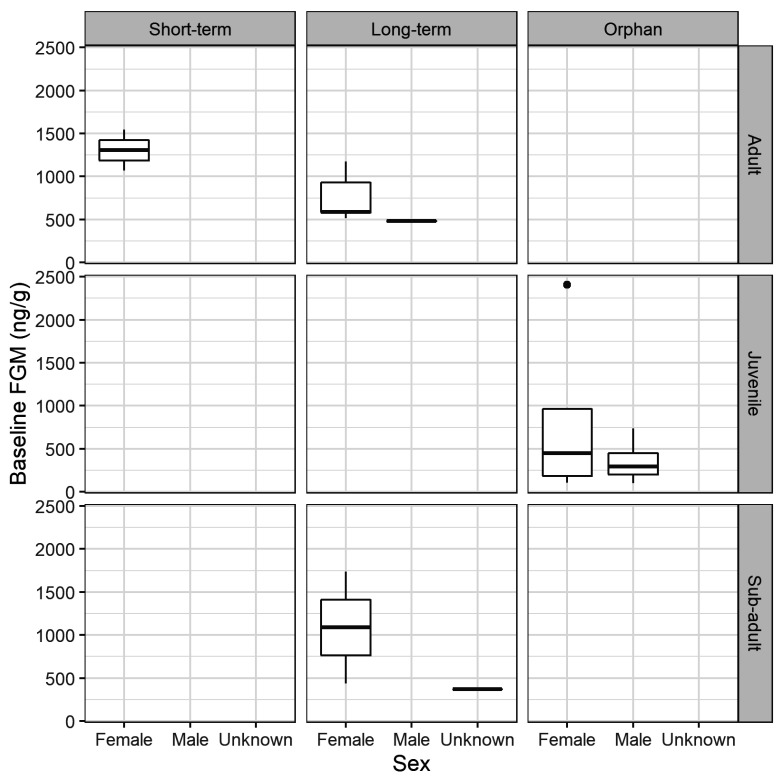
Boxplots of baseline faecal glucocorticoid metabolite (FGM) concentration thresholds (ng/g; mean plus 1.5 SD of baseline concentrations) by sex (female *n* = 14, male *n* = 5, unknown *n* = 1), length of stay (short-term *n* = 2, long-term *n* = 9, orphan *n* = 9) and age group (adult *n* = 8, juvenile *n* = 9, sub-adult *n* = 3) for possums in rehabilitation. Boxplots depict the median (dark line), two hinges (first and third quartiles), two whiskers (extending from the hinges to the smallest and largest values no further than 1.5 × inter-quartile range) and dots (outlying values).

Individual baseline threshold FGM concentrations ranged from 100 ng/g (SD 306, mean baseline values = 53 ng/g) to 2409 ng/g (SD 1697; mean baseline values = 1247 ng/g), with a mean of 748 ng/g (SD 599) for the 20 possums. There were no significant effects on baseline threshold of: short-term or long-term rehabilitation for adult females (F_(1,5)_ = 4.97, *p* = 0.076); adult or sub-adult age of long-term females (F_(1,5)_ = 0.667, *p* = 0.451); male or female sex for orphan juveniles (F_(1,7)_ = 0.878, *p* = 0.380); short-term, long-term or orphan rehabilitation for females (F_(2,11)_ = 0.411, *p* = 0.673); or adult, sub-adult or juvenile age for females (F_(2,11)_ = 0.106, *p* = 0.900) (Figure 3).

#### 3.2.3. Physiological Response to Potential Stressors

Eighteen of the 20 possums had increases in FGM concentrations (above baseline) after entering rehabilitation. Of these, 11 possums had peaks that occurred within five days of recorded potential stress events. Some possums expressed an FGM response soon after entry into rehabilitation (*n* = 6 of 15), the cover of the cage being removed (*n* = 1 of 1), being moved to a new cage (*n* = 4 of 5), a new possum entering the same cage (*n* = 1 of 2), handling (*n* = 2 of 2), being separated (1 of 2), and receiving an injection (*n* = 4 of 8; Table 2). There was no response to back riding (*n* = 0 of 1), escape from the cage (*n* = 0 of 1) or receiving oral medication (*n* = 0 of 2; Table 2; excluding possum 8295 given daily oral medication). There was no effect of the event type (Enter Rehabilitation versus Other Events) on the probability of an FGM response (LR χ^2^= 0.160, d.f. = 1, *p* = 0.689). The mean probability of an FGM response occurring within 5 days of an event was 47.7% (s.e. 7.4%).

**Figure 4:**
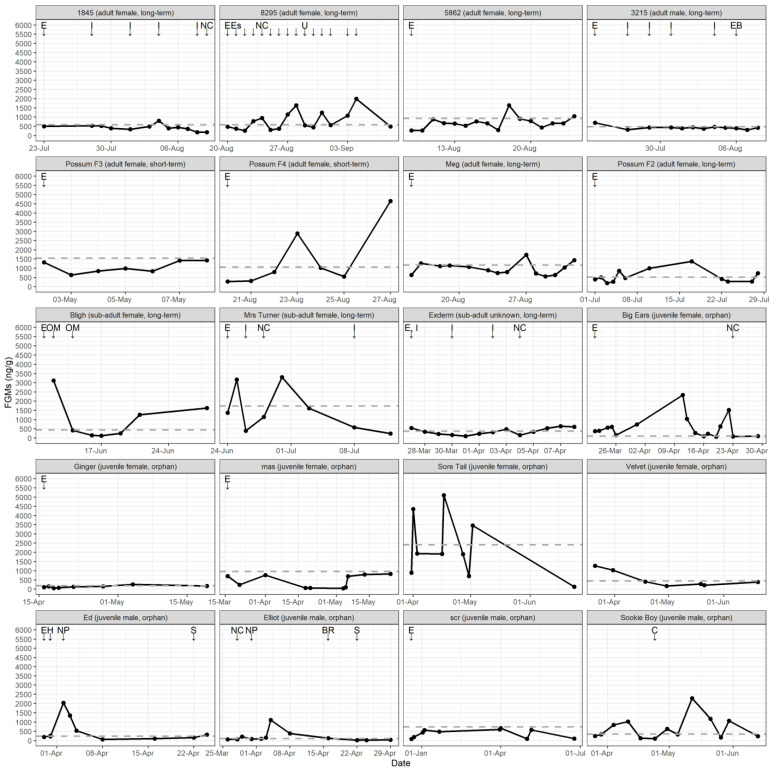
Faecal glucocorticoid metabolite (FGM) concentrations (ng/g) for 20 possums during rehabilitation. Potential stress events are represented as arrows, indicating when they occurred relative to sample collection (E = Enter rehabilitation; C = No cover; NC = New cage; NP = New possum; BR = Back rider; H = Handling; S = Separated; I = Injection; OM = Oral medication; EB = excited behaviour and movements; Es = escaped cage; U = a lot of urine noticed in cage; possum 8295 received daily oral medication in addition to labelled events). The grey dashed line represents the baseline threshold above which values are classified as peaks.

There were no consistent patterns in FGM concentrations for possums in relation to age, sex, or type of rehabilitation (Figure 4). Three sub-adult possums in long-term rehabilitation, named Bligh, Mrs Turner and Exderm, were diagnosed with stress dermatitis. Bligh and Exderm both showed a decrease in FGM concentrations from above baseline after entry into rehabilitation, however Mrs Turner had a more erratic pattern after entry to rehabilitation and one other possum (3215) without dermatitis also showed a decrease from above baseline after entering rehabilitation (Figure 4).

## 4. Discussion

This study has successfully validated an EIA for measuring FGMs in common brushtail possums and detected changes in FGMs during captivity and rehabilitation. Potentially stressful stimuli did not predictably elicit an increase in FGM in all possums, and the variable responses were likely influenced by the range in causes for rescue, presence of pre-existing diseases, the time between the onset of ailment and entry into rehabilitation, the captive environment, and individual personality [65].

All EIAs except R4866 detected peaks in FGMs in possums with sufficient accuracy, meeting the criteria for success by detecting a peak in FGMs post-ACTH challenge in a minimum of 50% of individuals (at least two of three possums in this case). Interestingly, the possum that was released from the study due to declining body condition showed a rapid increase in FGM of a large magnitude, suggesting that it was not coping with the captive environment. The sample size of three possums present for the validation process was sufficient for selecting an appropriate EIA but limited our ability to assess a wide range of individual animal responses. Similarly, few individuals per species were used in Fanson, et al. [37] to effectively select the most appropriate EIA for measuring FGM in a range of marsupial species, yet the authors acknowledged limits in assessing the effects of sex, reproductive state, and individual circumstances due to the small sample sizes. Typically, multiple individuals of both sexes should be included in a validation study to effectively assess the performance of an EIA [34,66]. Despite only having one male and two female possums for the entire ACTH challenge, expected FGM responses were detected allowing the validation process to be satisfactorily completed. By calculating the baseline threshold using the first sample plus 1.5 SD we were able to detect FGM peaks, despite collecting samples during a relatively short time in captivity. A validation of this assay will facilitate future research on the effects of age, sex and reproductive status in this species.

The 72a EIA was selected as the best option for further study of the GC response to rehabilitation in brushtail possums as it measured the greatest range of FGM concentrations and expressed the clearest increase and peak in FGMs in response to the ACTH challenge. The 72a EIA was the only assay to detect peaks in all three possums, likely because it is a group-specific assay, and these have been found to yield a stronger signal than cortisol or corticosterone assays [34]. A recent study also found that the 72a EIA could detect basal and peak FGM concentrations in marsupials including the northern hairy-nosed wombat (*Lasiorhinus krefftii*), yellow-bellied glider (*Petaurus australis)*, long-nosed potoroo (*Potorous tridactlus)*, woylie (*Bettongia penicillata*), southern bettong (*B. gaimardi*), eastern grey kangaroo (*Macropus giganteus)* and western grey kangaroo (*M. fuliginosus)* [37]. The 72a FGM concentrations ranged from 16 to 1347 ng/g (median 172 ng/g) in the ACTH challenge possums, and from 14 to 5105 ng/g (median 482 ng/g) in the possums admitted for rehabilitation. There are no existing baseline glucocorticoid data available for common brushtail possums to act as a comparison, and baselines are known to vary greatly between individuals, so it is most useful to inspect the magnitude of change existing in individual hormone profiles.

The possums involved in the ACTH challenge were exposed to both a natural stressor (capture and captivity) and a synthetic stressor (injection of exogenous ACTH) concurrently. Natural stressors are known to increase FGMs in wildlife, for example, following isolation and movement, baseline corticosterone of tammar wallabies (*Notamacropus eugenii*) increased by between 59% and 226% [67], and FGMs in Western lowland gorillas (*Gorilla gorilla gorilla*) increased in response to relocation, health checks, and introduction to other animals [68]. Possums were therefore expected to experience a significant increase in circulating glucocorticoids soon after capture and injection and the resulting peak in FGMs would be an indication of the excretion lag time (as injection of ACTH occurred minutes after capture), although a higher sampling frequency would be required to calculate an exact lag time.

The FGM peak times post ACTH challenge were highly variable across EIAs, even for the same individual. As faecal samples were only collected up to twice daily, the exact time between defecation and collection was unknown. In koalas, there was no effect of time since defecation for up to seven days for either faecal cortisol or corticosterone metabolite concentrations [69]. So, although the magnitude of the response may not have been affected by collecting samples several hours after defecation, an exact excretion lag time cannot be determined from our study. Nevertheless, based on the results (Table 1, Figure 2), the lag appears to be around 2–3 days. Faecal glucocorticoid metabolite excretion lag times in other species have ranged from seven hours in Columbian ground squirrels (*Spermophilus columbianus*; with a high frequency of sampling) [70] to 24 and 48 h in female and male koalas (with daily sampling) respectively [40]. An IV injection of cortisol in koalas resulted in a lag time of around 10 h, which more closely simulates the increase in cortisol in the plasma after a stressful event than the lag after an intramuscular ACTH injection [41]. A previous study of brushtail possums in New Zealand showed that radio-labelled particles and fluid were most active in the colon 24 to 32 h after dosing [71]. Therefore, FGMs were expected to be excreted somewhere within this range, and the lag time (shown as a significant rise in FGM post-ACTH injection) detected by EIAs in our study support this estimate. Further studies are needed, with samples collected more frequently, to determine the FGM excretion time in brushtail possums more precisely.

The initial response to entry into rehabilitation could differ depending on the possum’s existing condition at the time of rescue, although there was too much variation in FGM responses in our study to draw any conclusions. There was a decrease from above baseline FGMs for one of eight adults without exudative dermatitis and two of three sub-adults with dermatitis, also known colloquially as stress dermatitis due to its possible association with environmental stressors [72,73] or in other species, stressors in captivity [74]. There is not enough evidence to show whether the decrease in glucocorticoids after entering rehabilitation was due to treatment of the possums’ ailments, acclimatisation to the temporary captivity or unrelated. Understanding how existing conditions will impact a possum’s response to rescue and rehabilitation will be useful for rehabilitators. In the current study, there was no sustained elevation of FGMs after capture in any of the possums. Instead, any increases in FGM were transient and returned to baseline values. This provides some evidence that rescue, captivity, and rehabilitation per se did not cause chronic stress for these possums. In a study of oiled little penguins (*Eudyptula minor*), there were no long-term effects of rehabilitation on adrenal responses to humans in comparison to wild penguins [75], and a study of translocated elephants (*Loxodonta africana*) showed that FGMs were elevated during translocation but returned to baseline levels within 23 days [76]. Acute stressors have little impact on the long-term fitness of an animal, however, repeated exposure to acute stressors can result in chronic stress [16,77] and concurrent stressors have been associated with an increasing number of mass mortality events [78]. For example, environmental stressors resulting in low leaf moisture availability, decreased tree availability, and low minimum temperatures caused increases in FGM concentrations of koalas with potential long-term effects on survival [79]. Therefore, it is important to minimise the duration and number of stressful stimuli, but also to recognise that some events can be stressful (or perceived to be stressful) to some individuals but not others.

The events that were recorded by rehabilitators as potential stressors were followed by an FGM response within five days in about 50% of cases. Previous studies have shown that captivity is associated with stressors for wildlife including capture and handling, social stress, and unfamiliar sensory stimuli [54,80,81]. However, we were unable to identify events that were consistently classified as stressors between and within individuals. The low sample size for all events other than Enter Rehabilitation and the resultant need to combine them into Other Events likely would have masked any differences in perceived severity of various stressors. There were also no trends in FGM response to events or baseline values between sexes, ages, or length of time in rehabilitation, although the sample size may have been too low to detect significant differences. The adrenal response could be moderated by factors such as individual possum personality [53,65,82], other context-specific factors such as predation risk [32,83], or the severity of the perceived threat over time [84,85], and so variations in response between individuals are not altogether surprising.

There is some evidence that holding wild animals in captivity can induce chronic stress [86] and therefore impair their ability to show a measurable response to a stressor [13]. Chronic stress can cause GCs to be elevated above normal baseline levels [87], but has also been shown to lead to hypocortisolism in some cases [88,89]. A review of studies on chronically stressed wild animals found that endocrine responses were not consistent between species, and the presence of a change is more important than the direction of the change [90]. Our study did not find any differences in FGM baselines between female possums in captivity for a short or long period of time (i.e., short-term vs long-term groups), yet this must be interpreted cautiously due to the small sample size. Similarly, a recent study of wild rodents (*Rattus norvegicus* and *Rattus rattus*) did not observe any chronic stress due to captivity [84], and a study of river otters (*Lontra canadensis*) found that FGM did not increase over the first 10–12 days in captivity [81]. Further to our small sample size, another confounding factor in detecting chronic stress is that we do not know how truly basal or “unstressed” these baselines can be considered without having samples prior to rescue, nor do we know how long they had been suffering from their ailment prior to entry into rehabilitation. For example, the possum Meg had an eye injury and infection, PossumF2 had a leg injury, Bligh, Mrs Turner and Exderm were suffering dermatitis, Ginger had an eye ulcer, and Sore Tail had a tail injury for an unknown period of time before rescue. Normal baseline GCs for brushtail possums have not yet been established, so we are unable to currently discern whether a non-response to a potential stressor is due to chronic stress impacting their ability to respond versus the possum not finding the stimuli stressful. A long-term period of illness or injury may have artificially elevated their baseline at entry into rehabilitation, making it difficult to detect differences between cohorts and to detect peaks within individuals.

Future studies can build on our dataset by increasing the timespan of sample collection, recruiting a larger sample size, assessing the effects of seasonality [80,91,92], and increasing the daily sampling rate to assess diurnal changes in FGM [93]. It would be particularly valuable to monitor the FGM changes in a group of possums with fewer confounding factors (i.e., in the same rehabilitation environment, over the same time period, and with similar diagnoses) to reveal any effects, if they exist, of age, sex, and type of rehabilitation. Ensuring samples are collected daily is important when the aim is to detect all peaks in FGM, as peaks can be present in only a single sample [37]. As such, it is possible that FGM peaks were missed for possums in rehabilitation where sampling did not occur daily. It would be beneficial to measure FGMs in wild non-rehabilitated possums to establish the natural variation observed in presumptive “healthy” undisturbed animals, taking account of age, sex, and seasonal variations. There are also likely to have been additional potential stressors (perceived or actual) that were not recognised by rehabilitators involved in our study, such as domestic animals approaching rehabilitation enclosures, loud noises, or the presence of bright lights at night. These variables could not be completely controlled, and participants were recruited based on availability. To expand our understanding of the impact of stress on rehabilitation success in Australia, future studies should focus on including other commonly rescued species, including eastern grey kangaroos and ringtail possums (*Pseudocheirus peregrinus*), and assessing the relationship between the physiological GC response during rehabilitation and post-release survival.

It is widely accepted that minimising stressful stimuli experienced by wildlife in captivity is important for their welfare. However, we need to determine what constitutes stressful stimuli for wildlife and whether this can change depending on the species, the context, or the characteristics of the individual. We have provided evidence that brushtail possums exhibit changes in FGMs during captivity and rehabilitation with a high level of individual variability, and our findings are a starting point for assessing the link with potentially stressful stimuli. We also need to know if there could be any disadvantages of removing potential stressors, such as reduced fitness after release. To reduce stressful stimuli, rehabilitators are trained to cover an animal’s head when handling it to keep it calm, decrease noise, use slow movements, and cluster activities to reduce the number of human interactions [94,95]. However, there is a need for evidence-based guidelines as we do not know which practices are most effective alone or in combination to reduce stress. For example, it is common practice to provide a quiet environment for wildlife [15,94], yet a study found that birds that had been exposed to background radio noise during rehabilitation had reduced startle responses than those kept with no noise [96]. Therefore, it may be beneficial to maintain a modicum of mildly stressful stimuli to ensure the development of normal acute stress responses both while in captivity and after release into the wild.

## 5. Conclusions

Our study has successfully identified a suitable EIA for measuring FGMs in common brushtail possum faeces and has detected physiological GC changes in possums in rehabilitation. We recommend that future studies examine how specific stressors affect physiological changes. We have developed a list of potentially stressful stimuli that preceded a transient rise in FGMs in captivity for some possums, and this builds upon previous work in other species [67,80]. We did not detect a chronic elevation in FGMs in response to captivity for rehabilitation, suggesting that possums can tolerate captivity and rehabilitation without long-term consequences on their physiology. However, further research is needed to confirm if this was a positive outcome, as hypocortisolism is a potential outcome of chronic stress and could mask the long-term effects of rehabilitation [88,89]. Our study is the first step in building a robust dataset to explore important questions relating to predictors of stress during rehabilitation. Wildlife rescue and rehabilitation is carried out around the world, and it is likely to increase in scale in the future. As such, it is vital to develop evidence-based guidelines to promote animal welfare and survival at all stages of rescue, rehabilitation, release, and post-release. Future research should assess whether changes in FGMs during the rehabilitation of possums and other commonly rescued species are linked to rehabilitation outcomes pre- and post-release.

## Figures and Tables

**Table 1:** Time from adrenocorticotrophin hormone (ACTH) administration to first faecal glucocorticoid metabolite (FGM) value above baseline and maximum FGM concentration, mean peak and baseline concentrations (ng/g), and magnitude change in FGM concentration from first sample (%) as detected in possums by AA-corticosterone, CJM006, 72a, R4866 and AA-cortisol enzyme immunoassays (EIAs; note: dashes represent cases where no FGM peak was detected). Note: Female 3 was removed from the study on Day 4 due to declining body weight.

	Adult Possum	72a	AA-Corticosterone	AA-Cortisol	CJM006	R4866
Time to first FGM rise above baseline threshold (and time to maximum FGM if different from first rise)	Female 1	55 h 0 min (105 h 30 min)	31 h 30 min	7 h 0 min	31 h 30 min	- (7 h 0 min)
	Female 2	55 h 27 min	55 h 27 min (200 h 40 min)	32 h 6 min	55 h 27 min (82 h 31 min)	32 h 6 min
	Female 3	31 h 25 min	- (31 h 25 min)	31 h 25 min	- (31 h 25 min)	- (31 h 25 min)
	Male	8 h 40 min (31 h 40 min)	31 h 40 min	- (8 h 40 min)	- (31 h 40 min)	- (31 h 40 min)
Mean baseline FGM concentration (ng/g)	Female 1	385	189	46	254	130
	Female 2	124	184	93	185	150
	Female 3	709	510	72	505	210
	Male	194	122	40	186	119
Mean peak FGM concentration (ng/g)	Female 1	1066	304	103	373	-
	Female 2	852	342	234	275	612
	Female 3	1187	-	199	-	-
	Male	372	363	-	-	-
Magnitude of change from first sample at peak FGM (%)	Female 1	1330	254	186	175	135
	Female 2	6277	314	262	167	461
	Female 3	264	140	229	110	142
	Male	454	168	100	109	150

**Table 2:** Number and type of events associated with a faecal glucocorticoid metabolite (FGM) response (indicated by a peak in FGM above the baseline threshold within five days of a recorded potential stressor) or no FGM response for brushtail possums in rehabilitation (*n* = 20).

Event	FGM Response	No FGM Response	Total Events
Enter rehabilitation	6	9	15
Cover removed	1	0	1
New cage	4	1	5
New possum	1	1	2
Back rider	0	1	1
Handling	2	0	2
Separated	1	1	2
Injection	4	8	12
Oral medication	0	2	2
Escaped	0	1	1

## Data Availability

The data presented in this study are available in the article and Supplementary Material.

## Supplementary Materials

The following are available online at https://www.mdpi.com/article/10.3390/ani12131627/s1, Figure S1: Faecal glucocorticoid metabolite (FGM) concentrations (ng/g) over 9 days for four possums used in the ACTH challenge, Table S1: Summary statistics for possum faecal glucocorticoid concentrations, iterative baseline and response to potential stress events, Table S2: Number of events associated with a FGM response.
