# Supplementary material for: Validation of an Enzyme Immunoassay to Measure Faecal Glucocorticoid Metabolites in Common Brushtail Possums (Trichosurus vulpecula) to Evaluate Responses to Rehabilitation
Source: Animals (Basel). 2022 Jun 24;12(13):1627. doi: 10.3390/ani12131627 (PMC9265043; doi:10.3390/ani12131627)
Supplement: Supplementary file 1 [file animals-12-01627-s001.zip › Figure S1 Final.pdf]

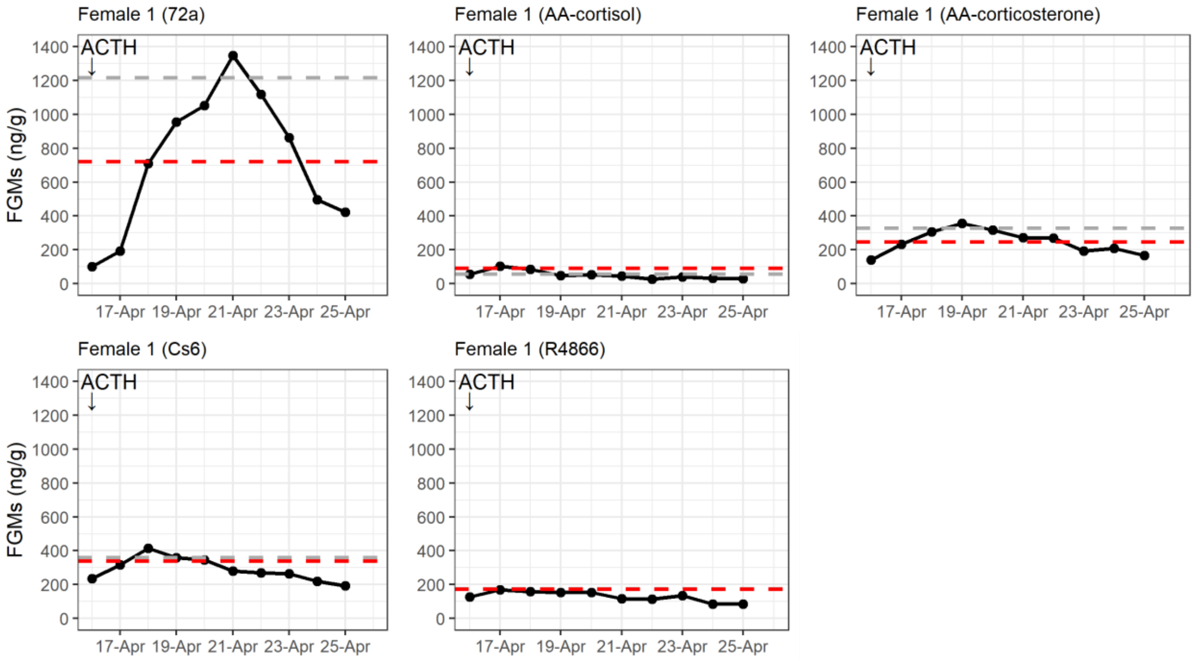

Female 1 (72a)
Female 1 (AA-cortisol)
Female 1 (AA-corticosterone)
Female 1 (Cs6)
Female 1 (R4866)
ACTH
FGMs (ng/g)
0
200
400
600
800
1000
1200
1400
17-Apr
19-Apr
21-Apr
23-Apr
25-Apr

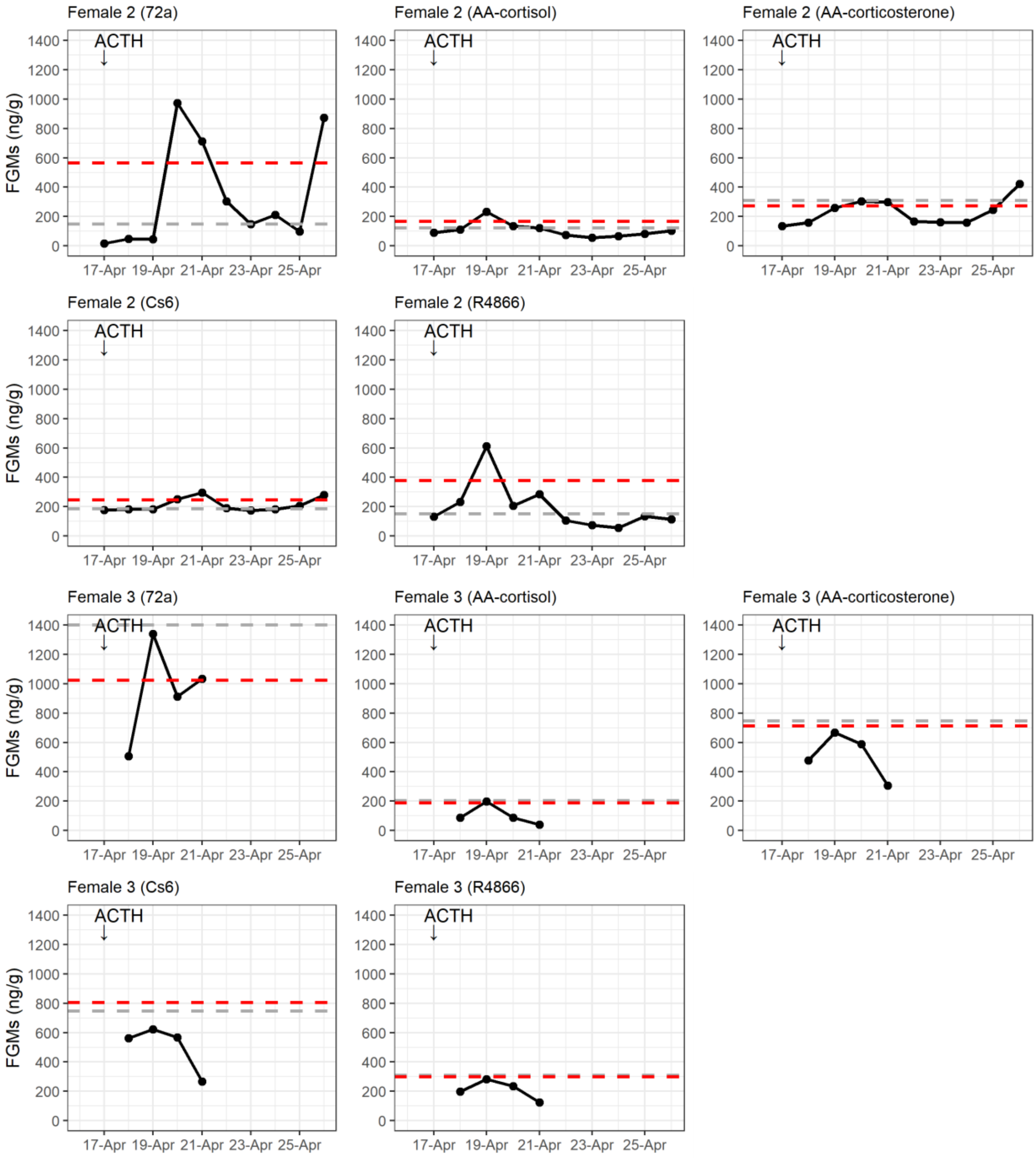

Female 2 (72a)
Female 2 (AA-cortisol)
Female 2 (AA-corticosterone)
Female 2 (Cs6)
Female 2 (R4866)
Female 3 (72a)
Female 3 (AA-cortisol)
Female 3 (AA-corticosterone)
Female 3 (Cs6)
Female 3 (R4866)
ACTH
FGMs (ng/g)
0
200
400
600
800
1000
1200
1400
17-Apr
19-Apr
21-Apr
23-Apr
25-Apr

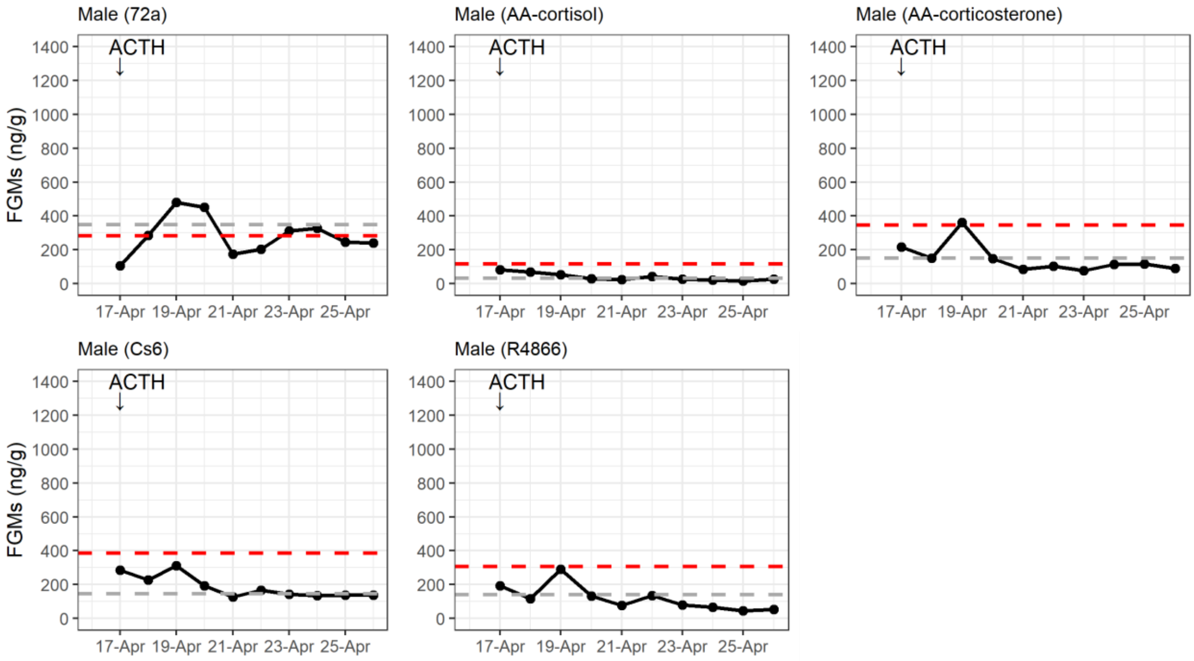

Figure S1. Faecal glucocorticoid metabolite (FGM) concentrations (ng/g) over 9 days for Female 1, Female 2, Female 3 and Male used in the ACTH challenge. Results depict FGM concentrations as detected by the enzyme immunoassays 72a, AA-cortisol, AA-corticosterone, Cs6 and R4866. Horizontal dotted lines represent the baseline threshold for the individual as calculated by the iterative baseline method (light grey) and the first sample plus 1.5 SD (red; the method used for validation analysis to detect biologically valid FGM peaks), and time of ACTH administration is indicated by the downward arrow.
