# Supplementary material for: Validation of an Enzyme Immunoassay to Measure Faecal Glucocorticoid Metabolites in Common Brushtail Possums (Trichosurus vulpecula) to Evaluate Responses to Rehabilitation
Source: Animals (Basel). 2022 Jun 24;12(13):1627. doi: 10.3390/ani12131627 (PMC9265043; doi:10.3390/ani12131627)
Supplement: Supplementary file 1 [file animals-12-01627-s001.zip › Table S1 Final.pdf]

Table S1. Summary statistics for possum (n = 20) faecal glucocorticoid metabolite (FGM) concentrations (ng/g), iterative baseline and response to potential stress events (E = Enter rehabilitation; Es = escaped cage; C = No cover; NC = New cage; NP = New possum; BR = Back rider; H = Handling; S = Separated; I = Injection; OM = Oral medication).

**Animal**	**Type**	**Sex**	**Age**	**Samples (n)**	**Mean FGM (ng/g)**	**Median FGM (ng/g)**	**S.D.**	**S.E.M.**	**CV %**	**Range**	**Baseline threshold**	**Mean baseline**	**Mean peak**	**Response to events**
1845	Long-term	Female	Adult	12	428	421	169	49	40	174 - 806	583	394	806	✓: I<br>✗: E, I, I
5862	Long-term	Female	Adult	16	697	662	339	85	49	272 - 1638	928	605	1342	✓:<br>✗: E
8295	Long-term	Female	Adult	16	792	563	507	127	64	268 - 1991	590	429	1260	✓: E, Es, NC, daily OMx15<br>✗:
3215	Long-term	Male	Adult	12	426	425	99	29	23	309 - 698	480	401	698	✓:<br>✗: E, I, I, I, I
PossumF3	Short-term	Female	Adult	7	1067	993	318	120	30	631 - 1427	1545	1067		✓: E<br>✗:
PossumF4	Short-term	Female	Adult	7	1500	794	1652	625	110	281 - 4649	1067	592	3770	✓: E<br>✗:
Meg	Long-term	Female	Adult	14	984	967	339	91	35	557 - 1727	1172	850	1477	✓:<br>✗: E
PossumF2	Long-term	Female	Adult	12	567	445	358	103	63	198 - 1380	518	354	993	✓: E<br>✗:
Bligh	Long-term	Female	Sub-adult	8	2358	838	3994	1412	169	125 - 3119	437	236	4480	✓:<br>✗: E, OM, OM
Mrs Turner	Long-term	Female	Sub-adult	8	1477	1256	1189	420	81	238 - 3303	1737	890	3240	✓: E, I, NC<br>✗: I
Exderm	Long-term	Unknown	Sub-adult	13	361	335	183	51	51	107 - 643	369	235	562	✓: I, I, NC<br>✗: E, I

Big Ears	Orphan	Female	Juvenile	16	575	382	615	154	107	71 - 2340	106	87	738	✓: E<br>×: NC
Ginger	Orphan	Female	Juvenile	8	138	138	63	22	45	59 - 263	181	121	263	✓:<br>×: E
mas	Orphan	Female	Juvenile	10	431	466	353	112	82	43 - 832	961	431		✓:<br>×: E
Sore tail	Orphan	Female	Juvenile	9	2266	1918	1697	566	75	135 - 5105	2409	1247	4304	N/A
Velvet	Orphan	Female	Juvenile	7	538	388	432	163	80	179 - 1269	447	293	1152	N/A
Ed	Orphan	Male	Juvenile	11	485	226	631	190	130	62 - 2043	231	138	903	✓: E, H, H, S<br>×: NP
Elliot	Orphan	Male	Juvenile	12	197	90	306	88	156	14 - 1112	100	53	398	✓: NC, NP<br>×: BR, S
scr	Orphan	Male	Juvenile	10	382	461	237	75	62	83 - 676	737	382		✓:<br>×: E
Sookie Boy	Orphan	Male	Juvenile	13	661	333	622	173	94	110 - 2287	354	221	1174	✓: C<br>×:
