# Supplementary material for: Validation of an Enzyme Immunoassay to Measure Faecal Glucocorticoid Metabolites in Common Brushtail Possums (Trichosurus vulpecula) to Evaluate Responses to Rehabilitation
Source: Animals (Basel). 2022 Jun 24;12(13):1627. doi: 10.3390/ani12131627 (PMC9265043; doi:10.3390/ani12131627)
Supplement: Supplementary file 1 [file animals-12-01627-s001.zip › Table S2 Final.pdf]

**Table S2.** Number of events associated with a faecal glucocorticoid metabolite (FGM) response (indicated by a peak in FGM above the baseline threshold within five days of a recorded stress event) or no FGM response for brushtail possums (*n*=20) in various length of rehabilitation, sex, and age categories.

| **Possum description** | **FGM response** | **No FGM response** | **Total events** |
|---|---|---|---|
| Short-term, female, adult (*n*=2) | 2 | 0 | 2 |
| Long-term, female, adult (*n*=5) | 5 | 6 | 11 |
| Long-term, male, adult (*n* = 1) | 0 | 6 | 6 |
| Long-term, female, sub-adult (*n*=2) | 3 | 4 | 7 |
| Long-term, unknown sub-adult (*n*=1) | 2 | 3 | 5 |
| Orphan, female, juvenile (*n*=5) | 1 | 3 | 4 |
| Orphan, male juvenile (*n*=4) | 7 | 4 | 11 |
